# Supplementary material for: Antisense oligonucleotide targeting the E3 ligase RFFL potentiates CFTR modulator efficacy in CF primary bronchial epithelial cells
Source: Mol Ther Nucleic Acids. 2025 Oct 31;36(4):102756. doi: 10.1016/j.omtn.2025.102756 (PMC12657287; doi:10.1016/j.omtn.2025.102756)
Supplement: Document S1. Figures S1–S3 and supplemental methods [file mmc1.pdf]

OMTN, Volume *36*

Supplemental information

Antisense oligonucleotide targeting the E3 ligase RFFL potentiates CFTR modulator efficacy in CF primary bronchial epithelial cells

Daichi Hinata, Yukari Kai, Ryosuke Fukuda, Yuka Kamada, Yuuya Kasahara, Kiyomi Sasaki, Tokuyuki Yoshida, Satoshi Obika, Takao Inoue, and Tsukasa Okiyoneda

Supplemental Methods

CFTR-Nluc degradation assay

CFBE Tet-on ΔF508-CFTR-3HA-Nluc cells were generated by lentiviral transduction using the pLIX vector as previously described.[10] Cells were seeded in 96-well plates and transfected with 20 nM ASO using Lipofectamine RNAiMax. Analyses were performed 4 days after transfection. CFTR expression was induced with 1 µg/mL doxycycline (Dox) for 4 days, and cells were treated with ETI (1 µM ELX, 3 µM TEZ, 1 µM IVA) for 2 days at 37° C. To minimize the contribution of immature ΔF508-CFTR during Nano-Glo Endurazine substrate loading, cells were treated with 100 µg/mL cycloheximide (CHX) for 3 hours at 37° C in CO_2-independent medium (ThermoFisher) according to the manufacturer's instructions. Following 3 hours of substrate loading, ΔF508-CFTR-Nluc luminescence was recorded continuously (every 10 min) at 37° C using a Luminoskan microplate reader (ThermoFisher). The degradation rate of ΔF508-CFTR-Nluc was calculated by fitting the luminescence decay curve with a one-phase exponential decay function in GraphPad Prism 8 (GraphPad Software).

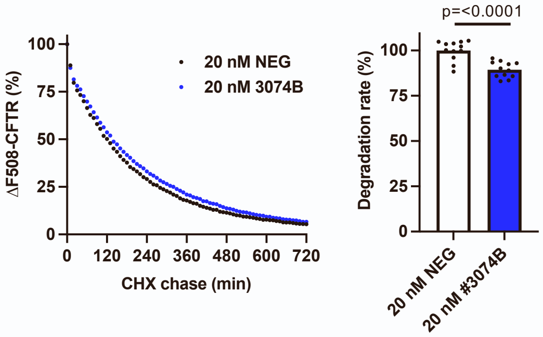

Figure S1.

Effect of RFFL ASO on mature ΔF508-CFTR-Nluc degradation in CFBE cells

Representative traces of ΔF508-CFTR-Nluc degradation in CFBE Tet-on cells transfected with 20 nM ASO are shown (left). Cells were pre-treated with ETI (1 µM ELX, 3 µM TEZ, 1 µM IVA) for 2 days at 37° C. To reduce the contribution of immature ΔF508-CFTR, cells were treated with CHX for 3 hours before measurement. The degradation rate of mature ΔF508-CFTR-Nluc was quantified (right). Statistical significance was assessed by unpaired t-test. Data represent mean values (n = 12).

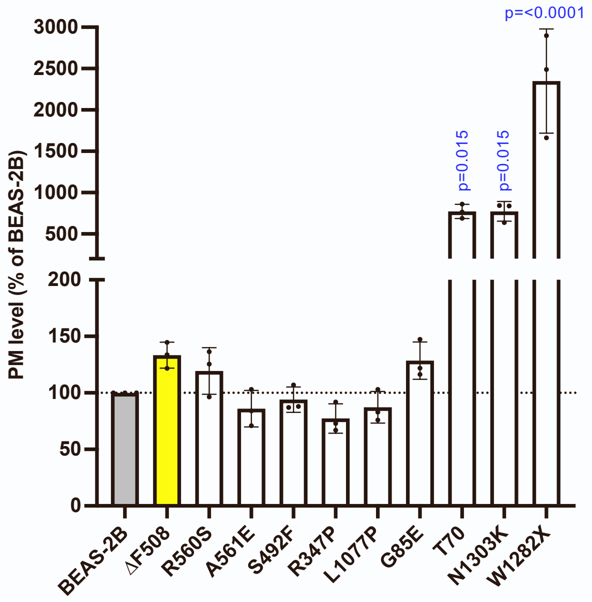

Figure S2.

PM levels of CFTR-HiBiT variants in BEAS-2B cells

Basal PM expression levels of CFTR-HiBiT mutants in BEAS-2B cells were quantified using the HiBiT assay and expressed as a percentage relative to parental BEAS-2B cells, which served as the background signal. Data are presented as mean ± SD (n = 3). Statistical significance was determined using one-way ANOVA followed by Dunnett's multiple comparison test. For data showing a significant effect, the corresponding *p*-value is indicated in the figure.

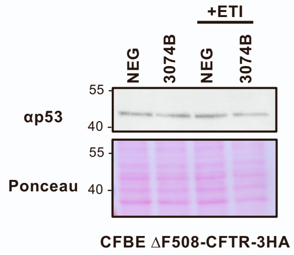

Figure S3.

Effect of RFFL ASO on p53 protein levels

Western blot analysis of ΔF508-CFTR-3HA in CFBE Tet-on ΔF508-CFTR-3HA cells transfected with 20 nM of either negative control ASO (NEG) or RFFL ASO 3074B (#37). Cells were treated with ETI (1 µM ELX, 3 µM TEZ, and 1 µM IVA) for 2 days at 37° C. The same lysates used in Fig. 3A were analyzed, and p53 was detected using an anti-p53 antibody (F16A10, Selleck, Cat#F0020). Ponceau staining was used as a loading control.
