# Supplementary material for: Antisense oligonucleotide targeting the E3 ligase RFFL potentiates CFTR modulator efficacy in CF primary bronchial epithelial cells
Source: Mol Ther Nucleic Acids. 2025 Oct 31;36(4):102756. doi: 10.1016/j.omtn.2025.102756 (PMC12657287; doi:10.1016/j.omtn.2025.102756)
Supplement: Document S2. Article plus supplemental information [file mmc2.pdf]

Molecular Therapy
Nucleic Acids
Original Article

Antisense oligonucleotide targeting the E3 ligase RFFL potentiates CFTR modulator efficacy in CF primary bronchial epithelial cells

Daichi Hinata,[1] Yukari Kai,[1] Ryosuke Fukuda,[1] Yuka Kamada,[1] Yuuya Kasahara,[2,3] Kiyomi Sasaki,[4] Tokuyuki Yoshida,[4] Satoshi Obika,[3] Takao Inoue,[4] and Tsukasa Okiyoneda[1]

[1]Department of Biomedical Sciences, School of Biological and Environmental Sciences, Kwansei Gakuin University, Hyogo 669-1330, Japan; [2]National Institutes of Biomedical Innovation, Health and Nutrition, Osaka 567-0085, Japan; [3]Graduate School of Pharmaceutical Sciences, The University of Osaka, Osaka 565-0871, Japan; [4]Division of Molecular Target and Gene Therapy Products, National Institute of Health Sciences, Kawasaki, Kanagawa 210-9501, Japan

Cystic fibrosis (CF) is most commonly caused by the ΔF508 mutation in the CFTR gene, leading to misfolding and degradation of the CFTR protein. Although CFTR modulators such as elexacaftor/tezacaftor/ivacaftor (ETI) provide clinical benefit, their efficacy is limited, particularly in patients with rare or poorly responsive CFTR mutations. RFFL, an E3 ubiquitin ligase, plays a central role in peripheral quality control of CFTR, reducing its plasma membrane (PM) expression and attenuating the effects of modulators. Here, we developed antisense oligonucleotides (ASOs) containing artificial nucleic acids to selectively suppress RFFL expression. An optimized *RFFL*-targeting ASO enhanced the efficacy of CFTR modulators by increasing the functional PM expression of ΔF508-CFTR in primary human bronchial epithelial (CF-HBE) cells derived from CF patients. Notably, the ASO also potentiated the effects of ETI on CFTR mutants associated with rare forms of CF, including those with limited responsiveness to modulators. In some cases, the ASO alone restored CFTR levels to those achieved by ETI treatment. These findings establish *RFFL*-targeting ASOs as first-in-class CFTR stabilizers and highlight their potential as a nucleic acid-based therapeutic strategy for CF caused by both common and rare CFTR mutations.

INTRODUCTION

Cystic fibrosis (CF) is a genetic disorder with high prevalence in Europe and the United States, affecting approximately 89,000 individuals worldwide.[1] Around 90% of CF patients carry the ΔF508 mutation in the cystic fibrosis transmembrane conductance regulator (CFTR) gene, which results in the deletion of a phenylalanine residue at position 508 in the CFTR protein.[2] Although ΔF508-CFTR is normally transcribed and translated, the mutation causes protein misfolding due to conformational defects. As a result, the misfolded protein is recognized by the endoplasmic reticulum quality control (QC) system, ubiquitinated, and subsequently targeted for degradation, thereby failing to reach the plasma membrane (PM).[2–4] In recent years, several CFTR modulators have been developed as breakthrough therapies for CF.[5–7] Among them, Trikafta, a triple combination therapy comprising elexacaftor ([ELX] VX-445), tezacaftor ([TEZ] VX-661), and ivacaftor ([IVA] VX-770), collectively referred to as ELX/TEZ/IVA (ETI), has shown the greatest clinical efficacy.[8,9] This regimen works synergistically to correct ΔF508-CFTR misfolding, promote its trafficking to the PM, and enhance its chloride channel function.

We previously demonstrated that the E3 ubiquitin ligase RFFL (ring finger and FYVE-like domain containing E3 ubiquitin protein ligase) plays a pivotal role in the degradation of CFTR at the PM.[10] Knockdown (KD) of RFFL suppresses the degradation of cell surface ΔF508-CFTR and enhances the efficacy of CFTR modulators such as ETI, thereby improving ΔF508-CFTR function.[10,11] These findings suggest that combining RFFL KD with CFTR-modifying therapies could lead to improved therapeutic outcomes for CF patients.[12,13] However, it remains unclear whether RFFL KD can enhance the function of endogenous ΔF508-CFTR when combined with clinically approved CFTR modulators in primary human bronchial epithelial (HBE) cells derived from CF patients homozygous for the ΔF508 mutation (CF-HBE). CF-HBE cells are considered the gold standard for investigating CF disease pathogenesis and evaluating CFTR modulator efficacy.[14,15] Furthermore, our earlier work revealed that RFFL KD alone, even in the absence of CFTR modulators, enhances PM expression of CFTR class VI mutants, including T70-CFTR, a rare mutation found in CF patients.[10] This observation indicates that RFFL KD may represent a promising therapeutic strategy for individuals with class VI CFTR mutations, independent of currently available CFTR modulators.[12]

Received 30 May 2025; accepted 28 October 2025;
https://doi.org/10.1016/j.omtn.2025.102756.

Correspondence: Tsukasa Okiyoneda, Department of Biomedical Sciences, School of Biological and Environmental Sciences, Kwansei Gakuin University, Hyogo 669-1330, Japan.
E-mail: t-okiyoneda@kwansei.ac.jp

© 2025 The Author(s). Published by Elsevier Inc. on behalf of The American Society of Gene and Cell Therapy.
This is an open access article under the CC BY license (http://creativecommons.org/licenses/by/4.0/).

Given these insights, we focused on antisense oligonucleotide (ASO) therapeutics as a promising modality for knocking down RFFL expression, to establish a novel therapeutic strategy for CF. ASO therapeutics can target RNA directly and have been developed to treat various diseases, including previously intractable human disorders.[16] Among these, gapmer ASOs exert their therapeutic effects by inducing the degradation of target RNA. Gapmer ASOs contain chemically modified ribose nucleotides in the flanking wing regions, which enhance RNA-binding affinity and confer nuclease resistance. Representative modifications include 2′-*O*-methoxyethyl-RNA (2′-MOE) and 2′,4′-bridged nucleic acids (BNAs),[17] such as locked nucleic acids (LNAs). The central gap region consists of natural deoxyribonucleotides. Gapmer ASOs bind to target RNA in a sequence-specific manner to form ASO-RNA heteroduplexes, which are recognized by RNase H1, resulting in the cleavage of the target RNA. So far, six 2′-MOE gapmer ASO therapeutics have been approved for clinical use.[18,19] Previously, we developed amido-bridged nucleic acids (AmNAs), a novel type of BNA. AmNAs exhibit superior nuclease resistance and structural stability compared to LNAs.[20] Building on this platform, we applied AmNA chemistry to design gapmer ASOs for the treatment of diseases such as cancers and central nervous system disorders and confirmed their low toxicity and potent therapeutic efficacy.[21–25]

In this study, we developed an AmNA gapmer ASO targeting *RFFL* (RFFL ASO), utilizing artificial nucleic acids and chemical modifications. The RFFL ASO was optimized for KD efficiency, enhancement of CFTR PM level, low cytotoxicity, and minimal induction of innate immune responses, an issue often associated with nucleic acid-based therapeutics. In a primary culture model of CF-HBE considered the gold standard for evaluating CF therapeutics,[14,15] we demonstrated that RFFL ASO effectively downregulates endogenous RFFL expression and improves ΔF508-CFTR function, thereby potentiating the efficacy of existing CFTR modulators. These findings position RFFL ASO as a promising first-in-class CFTR stabilizer that can potentiate the efficacy of CFTR modulators, and a potential therapeutic agent for CF patients with class VI CFTR mutations including ΔF508-CFTR and rare mutants.

RESULTS

Discovery of RFFL ASO

We initially aimed to design a chemically modified ASO capable of effectively suppressing RFFL expression. The ASO was constructed using a gapmer design that incorporated five AmNAs, and all internucleotide phosphodiester (PO) bonds were substituted with phosphorothioate (PS) linkages (Figure 1A, bottom).

The design of 28 candidate 15-mer gapmer ASOs targeting *RFFL* mRNA were informed by the following considerations. In accordance with the intramolecular secondary structure predicted by RNAfold (http://rna.tbi.univie.ac.at/cgi-bin/RNAWebSuite/RNAfold.cgi)[26] and UNAfold (https://www.unafold.org/),[27] non-stem regions were extracted from all regions of the *RFFL* mRNA (GenBank: NM_001017368.2). Among the ASO sequences complementary to the extracted regions, ASO candidates with toxic risk motif sequences such as TCC, TGC,[28] and unmethylated cytosine-phosphate-guanine (CpG) motifs[29] were eliminated. Subsequently, we selected ASO candidates with an appropriate binding affinity to RNA predicted by GC%, and ASOs themselves are unlikely to form intramolecular stems or homodimers. In addition, we excluded ASO candidates with high off-target risk[30] based on the results of the analysis using GGGenome (https://gggenome.dbcls.jp/) and Database for Drug Development with Genome and RNA sequences (https://d3g.riken.jp/). These ASO candidates were then evaluated based on three criteria: (1) the ability to reduce *RFFL* mRNA expression, (2) the ability to enhance ΔF508-CFTR PM expression, and (3) the absence of cytotoxic effects. ASOs satisfying all three criteria were identified as potential CFTR stabilizers.

To identify ASOs capable of effectively reducing *RFFL* mRNA levels, we transfected RFFL-ASOs into HeLa and 293MSR cells using the CEM (calcium enrichment of medium) method[31] and quantified *RFFL* mRNA expression by qPCR. This screening yielded multiple ASOs with KD efficiencies of 80% or higher in both HeLa and 293MSR cells (Figure 1B). In contrast, the CEM method failed to achieve high KD efficiency in CFBE Tet-on cells stably expressing ΔF508-CFTR-HRP (CFBE-ΔF508-CFTR-HRP) cells, whereas lipofection proved effective (Figure 1B). Therefore, lipofection was used for ASO delivery in CFBE cells in subsequent experiments. The cause of CEM transfection failure in CFBE cells remains unclear. However, based on our empirical observations, the CEM method tends to be less effective in cells with slow proliferation rates or relatively large cell sizes. Since CFBE cells proliferate slowly and are comparatively large, these features likely contribute to the poor transfection efficiency observed with the CEM approach in this cell line. Further evaluation of the CFBE airway epithelial cell lines derived from CF patients identified four ASOs (#14, #19, #20, and #25) with KD efficiencies exceeding 70%, achieved using lipofection with Lipofectamine RNAiMAX as the transfection reagent (Figure 1B).

Next, we evaluated the impact of RFFL-ASOs on ΔF508-CFTR PM expression. RFFL-ASOs were introduced into CFBE-ΔF508-CFTR-HRP via lipofection, and horseradish peroxidase (HRP) assays were performed as previously described.[10] Following transfection, the cells were incubated at 26°C for 2 days to promote ΔF508-CFTR PM expression, followed by a 1-h incubation at 37°C before the HRP assay to induce RFFL-mediated peripheral degradation.[10] As expected, several ASOs, including ASO#19, ASO#20, ASO#21, and ASO#25, significantly increased ΔF508-CFTR PM levels (Figure 1C). Cytotoxicity was also assessed using the AlamarBlue assay. While some ASOs, such as ASO#15, ASO#17, ASO#25, and ASO#28, exhibited cytotoxic effects, ASO#20 and ASO#21 demonstrated minimal toxicity (Figure 1D). Based on these results, we focused on ASO#20 as a promising candidate due to its low toxicity, efficient RFFL KD, and robust enhancement of CFTR PM expression in CFBE cells.

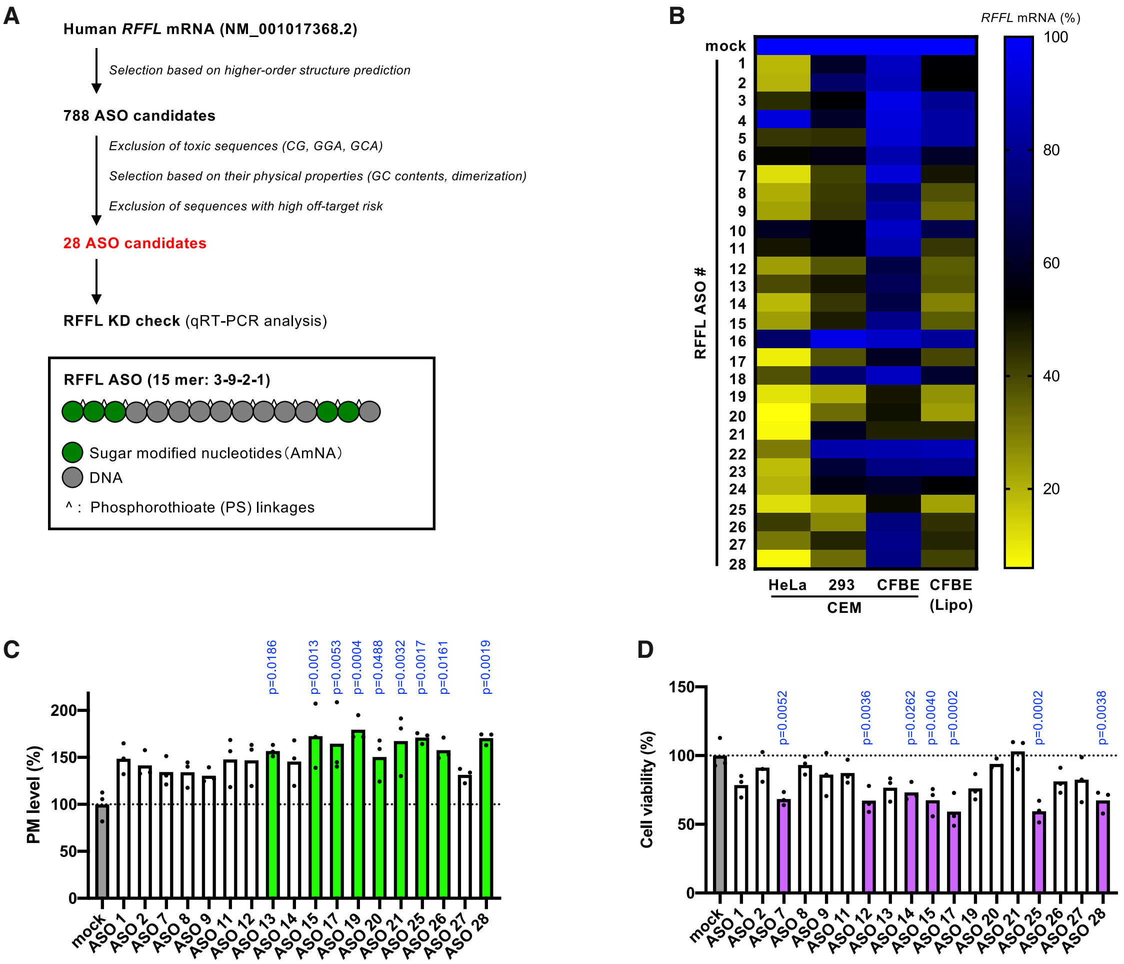

Figure 1. Selection of RFFL ASOs that enhance ΔF508-CFTR PM expression

(A) Schematic overview of the RFFL ASO screening workflow. (B) KD efficiency of RFFL ASOs in HeLa, 293MSR (293), and CFBE-ΔF508-CFTR-HRP (CFBE) cells was assessed by qPCR. RFFL ASO was delivered into HeLa, 293MSR, and CFBE cells using either the CEM method (100 nM) or lipofection (20 nM, Lipo), as specified. *RFFL* mRNA expression levels are presented as a heatmap ($n = 1$). (C, D) Effects of RFFL ASOs (20 nM, introduced via lipofection) on the PM expression of ΔF508-CFTR (C, $n = 3$) and cell viability (D, $n = 3$) in CFBE-ΔF508-CFTR-HRP cells. PM expression of ΔF508-CFTR was induced by incubation at 26°C for 2 days, followed by 1-h incubation at 37°C prior to analysis. Cell viability was measured by alamarBlue assay. Data represent mean values. Statistical significance was determined by one-way ANOVA with Dunnett's multiple comparison test. For data showing a significant effect, the corresponding p value is indicated in the figure.

Optimization of RFFL ASO

To further optimize RFFL ASOs for greater efficacy and safety, we designed new ASOs targeting regions adjacent to the ASO#20 target sequence, as well as an extended version of ASO#20 with the target sequence length increased from 15-mer to 17-mer (Figure 2A). Furthermore, we designed ASOs in which the PS of ASO#20 was partially replaced with PO (Figure 2B).

qPCR analysis revealed that while all tested ASOs exhibited strong KD efficiency in HeLa cells, only ASOs #31 and #34 showed modest KD activity in CFBE cells (Figure 2C). This suggests that the 5′ and 3′ end regions of the target sequence are critical for achieving high KD efficiency. Consistent with these results, most ASOs significantly increased the PM expression of ΔF508-CFTR with minimal cytotoxicity in CFBE cells (Figures 2D and 2E). Notably, ASOs #36, #37, and #38, designed with the same core sequence as ASO#20, robustly reduced *RFFL* mRNA levels and enhanced PM expression of ΔF508-CFTR (Figures 2C and 2D). Among these, ASO#37 (designated 3074B), which has two fewer PS linkages compared to ASO#20, demonstrated the best overall performance in terms of ΔF508-CFTR PM enhancement and low cytotoxicity, identifying it as the most promising RFFL ASO candidate (Figure 2F).

RFFL ASO enhances the efficacy of CFTR modulators in CFBE cell lines

To determine whether RFFL ASO 3074B enhances the therapeutic efficacy of clinically approved CFTR modulators, we evaluated its

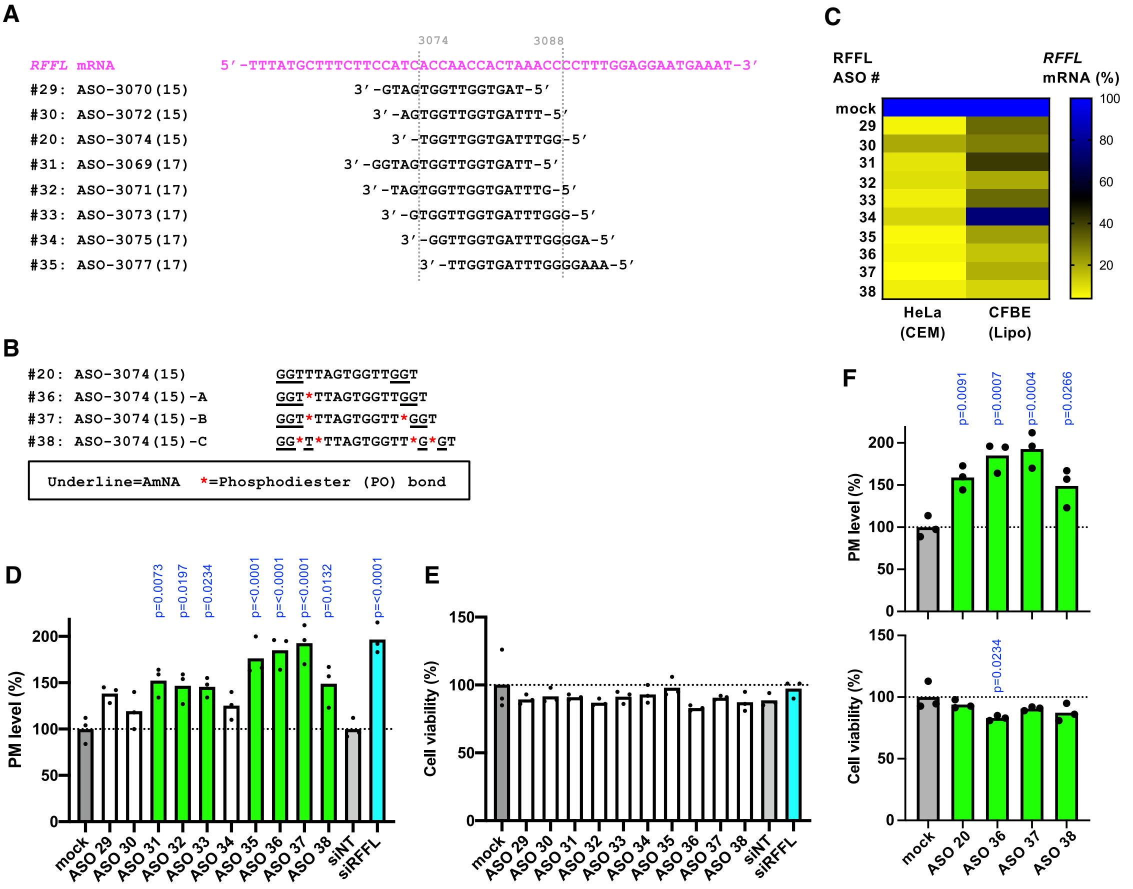

Figure 2. Optimization of RFFL ASO

(A) Target sequences of RFFL ASOs (#29–35) on the human *RFFL* mRNA are shown. (B) Sequence information of RFFL ASO (#20, #36–38) with various chemical modifications. Regions differing from ASO#20 are highlighted in red. (C) KD efficiency of optimized RFFL ASOs was evaluated by qPCR as described in Figure 1B (n = 1). (D and E) Effects of optimized RFFL ASOs on ΔF508-CFTR PM expression (D, n = 3) and cell viability (E, n = 3) in CFBE-ΔF508-CFTR-HRP cells. Cells were transfected with 20 nM ASO via lipofection, incubated at 26°C for 2 days to induce CFTR PM expression, and further incubated at 37°C for 1 h before analysis. Non-targeting small interfering RNA (siRNA) ([siNT] 50 nM) and RFFL-targeting siRNA ([siRFFL] 50 nM) were used as negative and positive controls, respectively. (F) Evaluations of ΔF508-CFTR PM expression (top, n = 3) and cell viability (bottom, n = 3) were conducted in CFBE-ΔF508-CFTR-HRP cells using the modified ASO#20 described in Figure 2B. Data represent mean values. Statistical significance was determined using one-way ANOVA followed by Dunnett's multiple comparison test (D–F). For data showing a significant effect, the corresponding p value is indicated in the figure.

effect on the triple combination therapy (ETI) using CFBE Tet-on cells stably expressing ΔF508-CFTR-3HA (CFBE-ΔF508-CFTR-3HA).[10] Western blot analysis revealed that ASO 3074B markedly increased the mature form of ΔF508-CFTR, but not the immature form, compared with the negative control ASO (NEG) in CFBE-ΔF508-CFTR-3HA cells treated with ETI (Figure 3A). In contrast, under ETI-free conditions, ASO 3074B had no significant effect on ΔF508-CFTR expression (Figure 3A). Consistently, the HRP assay demonstrated a significant increase in cell surface ΔF508-CFTR-HRP levels in cells treated with ASO 3074B compared to those treated with the NEG ASO, without inducing cytotoxicity (Figures 3B and 3C). Furthermore, cell surface ELISA analysis[32] demonstrated that ASO 3074B significantly increased the amount of ΔF508-CFTR remaining at the cell surface after a 2-h incubation, indicating improved cell surface stability (Figure 3D). Consistently, ASO 3074B also reduced the degradation of mature ΔF508-CFTR-Nluc in CFBE cells, an effect that closely paralleled our previous findings with RFFL small interfering RNA (Figure S1).[11] We also assessed ΔF508-CFTR channel function using a halide-sensitive yellow fluorescent protein (YFP) quenching assay.[10] ASO 3074B did not enhance channel activity in the absence of ETI, but it significantly increased channel function when combined with ETI (Figure 3E). Taken together, these findings suggest that ASO 3074B augments the therapeutic efficacy of ETI, most likely by suppressing RFFL-mediated degradation of ΔF508-CFTR at the PM, thereby increasing both its surface expression and channel activity.

Some ASOs, particularly those containing CpG motifs, can trigger an innate immune response by activating Toll-like receptor 9 (TLR9).[33] Although RFFL ASO 3074B was designed without unmethylated

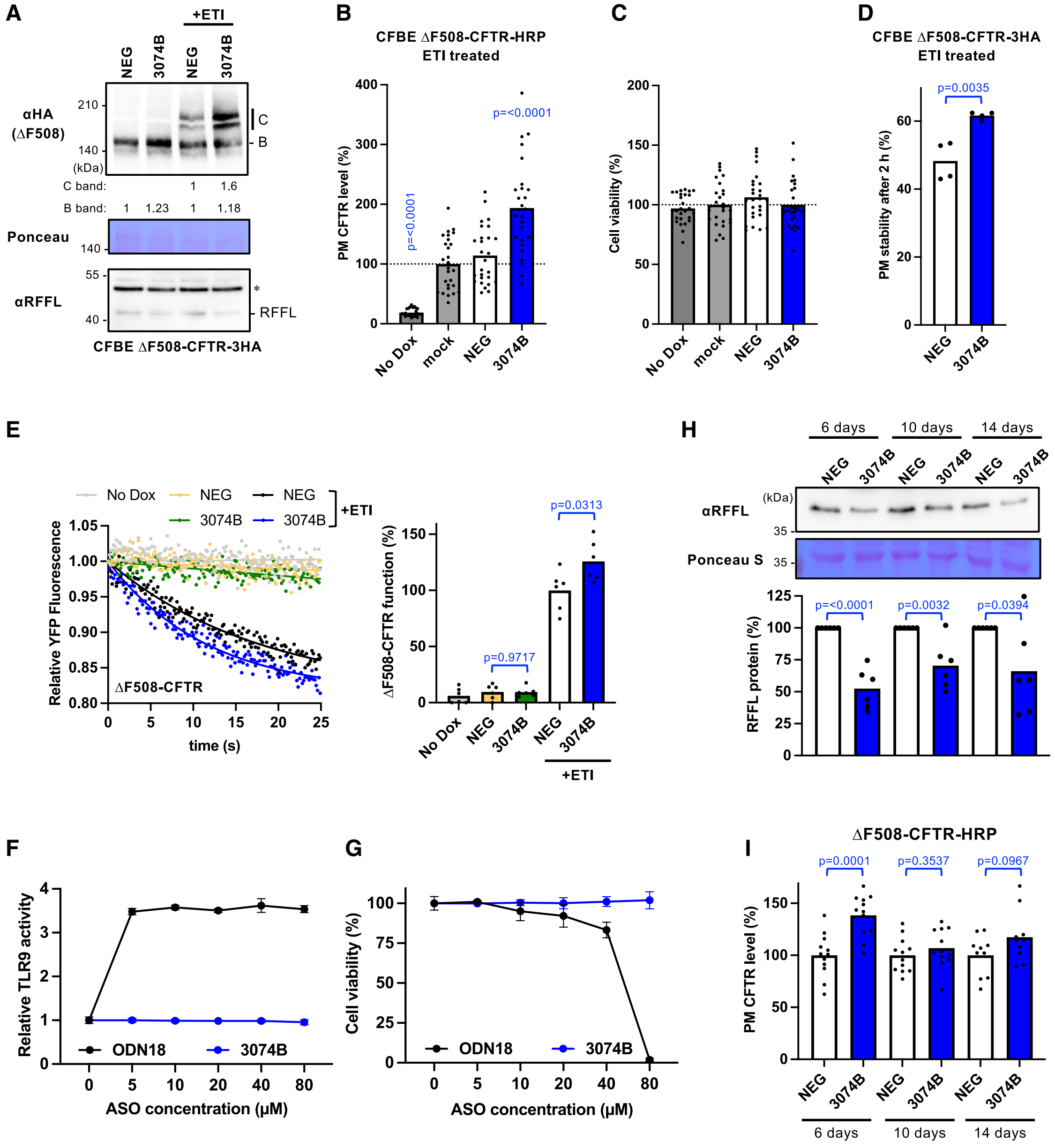

Figure 3. RFFL ASO 3074B improves the efficacy of CFTR modulators in CFBE cells

(A) Western blot analysis of ΔF508-CFTR-3HA in CFBE Tet-on cells transfected with 20 nM negative control ASO (NEG) or RFFL ASO 3074B (#37). Cells were treated with either 0.3% DMSO or ETI (1 μM ELX, 3 μM TEZ, 1 μM IVA) for 2 days at 37°C. The immature (B and B) and mature (B and C) forms of CFTR were quantified and normalized to NEG. Ponceau staining served as a loading control. The asterisk denotes a non-specific band. (B and C) Effects of RFFL ASO 3074B on ΔF508-CFTR PM levels (B, $n = 27$) and cell viability (C, $n = 27$) in CFBE-ΔF508-CFTR-HRP cells following lipofection with 20 nM ASOs. ΔF508-CFTR PM expression was induced by ETI treatment for 2 days at 37°C, as shown in Figure 3A. (D) PM stability of ΔF508-CFTR-3HA in CFBE Tet-on cells transfected with either NEG or RFFL ASO 3074B ($n = 4$). Cells were treated with ETI for 2 days at 37°C prior to analysis, as shown in Figure 3A. (E) The channel function of ΔF508-CFTR-3HA in CFBE Tet-on cells transfected with 50 nM NEG or ASO 3074B was assessed using the halide-sensitive YFP quenching assay. The initial YFP quenching rate was quantified as CFTR channel activity (right, $n = 6$). CFTR expression was induced by Dox (1 μg/mL) treatment for 4 days, and cells were pre-treated with ETI for 2 days at 37°C prior to the assay, as described in Figure 3A (+ETI). (F and G) TLR9-stimulatory activity and cell viability of RFFL ASO 3074B in HEK-Blue hTLR9 cells. HEK-Blue hTLR9 cells were treated with RFFL ASO 3074B, or the positive control SY-ODN18 (ODN18), at various concentrations for 18 h. TLR9 activity was then measured and normalized to the untreated control (F). Cell viability was assessed by the WST-8 assay and similarly normalized (G). Data are presented as mean ± SD ($n = 3$). (H) Western blot analysis demonstrates sustained RFFL KD following a single transfection of RFFL ASO in CFBE-ΔF508-CFTR-3HA cells. Cells were transfected with 20 nM of the indicated ASO. Endogenous RFFL protein levels were quantified by densitometry and expressed as a percentage relative to NEG ASO-transfected cells ($n = 6$). Ponceau staining served as a loading control. (I) PM levels of ΔF508-CFTR-HRP in CFBE Tet-on

(legend continued on next page)

CpG motifs, previous studies have shown that certain Non-CpG ASOs can still activate innate immune responses via TLR9.[34,35] Accordingly, we quantitatively assessed the TLR9-stimulatory potential of RFFL ASO 3074B as part of its safety evaluation, using human TLR9-expressing HEK-Blue hTLR9 cells, which secrete secreted embryonic alkaline phosphatase (SEAP) upon activation of the TLR9-NF-κB (nuclear factor-κB) signaling pathway.[36] As a positive control, we used SY-ODN18, a synthetic single-stranded oligodeoxynucleotide that we previously identified and confirmed to exhibit sufficient TLR9-stimulatory activity.[36] In contrast to SY-ODN18, ASO 3074B did not induce TLR9 activation (Figure 3F) or cytotoxicity at any of the concentrations tested (Figure 3G), thereby supporting the safety profile of ASO 3074B.

Furthermore, we assessed the duration of RFFL KD induced by a single treatment of RFFL ASO in CFBE cells. Western blot analysis demonstrated that a significant reduction in RFFL protein levels was sustained for up to 2 weeks following a single ASO administration (Figure 3H). We next examined whether the CFTR improvement induced by RFFL ASO was sustained over time by measuring cell surface levels of ΔF508-CFTR-HRP. The HRP assay revealed that elevated levels of cell surface ΔF508-CFTR were still detectable at 6 days post-transfection, but this effect was no longer evident by day 10 (Figure 3I). Thus, while a single ASO administration can maintain RFFL KD for approximately 2 weeks, the functional improvement in CFTR stability appears to last for about 1 week.

RFFL ASO enhances the efficacy of CFTR modulators in human CFTR$^{\Delta F508/\Delta F508}$ bronchial epithelia

Finally, we evaluated the add-on effect of ASO 3074B in combination with ETI treatment using human-derived tissue. Primary HBE cells from three individuals with CF, homozygous for the ΔF508 mutation (CF-HBE), were differentiated at the air-liquid interface (ALI). This CF-HBE model is widely recognized as the gold standard for assessing the efficacy of CF therapies.[14,15] CFTR function was quantified by measuring short-circuit current (Isc) stimulated by forskolin, with the CFTR inhibitor 172 (Inh-172)-sensitive component defined as CFTR-mediated current.[37] ASO 3074B was applied apically in hypo-osmotic saline and re-administered in isotonic saline; the solution was fully absorbed within 4 days, at which point endogenous ΔF508-CFTR function was assessed. ETI (3 μM ELX, 18 μM TEZ, 1 μM IVA) was applied from the basolateral side for 2 days at 37°C, under conditions similar to those described in previous studies.[9] As expected, ASO 3074B significantly enhanced endogenous ΔF508-CFTR function upon ETI treatment in CF-HBE cells derived from three CF patients (Figures 4A and 4B). RFFL KD was also confirmed by RT-qPCR in CF-HBE cells (Figure 4B). These results demonstrate that ASO 3074B effectively reduces RFFL expression and, consequently, enhances the efficacy of ETI by increasing cell surface ΔF508-CFTR levels, even in human-derived tissues.

Add-on effects of RFFL ASO with CFTR modulators across various CFTR mutants

Although approximately 90% of CF patients carry at least one allele of the ΔF508 CFTR mutation, numerous other CFTR mutations have been identified and are classified into six classes (I–VI) based on their functional defects.[38] For some of these classes, the impact of Trikafta (ETI treatment) appears to be less effective compared to ΔF508-CFTR.[39,40] Since the mechanism of action of ETI differs from that of RFFL ASO, RFFL ASO may have a beneficial effect on CFTR mutants that are less responsive to ETI. In particular for class VI CFTR mutants with low PM stability,[38] RFFL KD could enhance the PM stability of CFTR mutants even without ETI. As a result, RFFL ASO alone may improve CFTR PM expression in these cases. To evaluate the broader applicability of ASO 3074B, we utilized HBE BEAS-2B cells stably expressing various CFTR mutants, each tagged with a HiBiT sequence fused to the extracellular domain.[41] PM expression levels of the mutant CFTRs following ASO 3074B transfection were quantified using the HiBiT assay.[41] ASO 3074B treatment significantly enhanced the PM expression of all tested CFTR mutants in the presence of ETI (Figure 5A). Notably, ASO 3074B synergistically enhanced the efficacy of ETI in CFTR mutants including ΔF508, R560S, A561E, S492F, L1077P, G85E, N1303K, and W1282X (Figure 5A). Moreover, ASO 3074B significantly increased the PM levels of R347P, T70, and N1303K CFTR mutants even in the absence of ETI (Figure 5A). Correlation analysis between the effects of ETI and ASO 3074B treatment on PM expression levels across various CFTR mutants revealed no correlation, supporting their distinct mechanisms of action (Figure 5B). In contrast, a strong correlation was observed between the effects of ETI alone and ETI in combination with ASO 3074B (Figure 5C), indicating additive or synergistic effects. These results suggest that while ASO 3074B can enhance CFTR PM expression in mutants with low sensitivity to ETI, its additive or synergistic effects are even more pronounced in CFTR mutants that exhibit high responsiveness to ETI.

We next assessed whether the increased PM levels of various CFTR mutants corresponded to functional activity using the halide-sensitive YFP quenching assay. As expected, ASO 3074B enhanced the channel function of both N1303K-CFTR and T70-CFTR in the presence of ETI (Figures 5D and 5E). In contrast, ASO 3074B had no detectable effect on these mutants in the absence of ETI, likely due to the limited sensitivity of the assay (Figures 5D and 5E). Together, these findings suggest that ASO 3074B can enhance the functional

cells transfected with 20 nM NEG or ASO 3074B were measured at 6, 10, and 14 days post-transfection. CFTR expression was induced with Dox (1 μg/mL) for 4 days, and ETI (1 μM ELX, 3 μM TEZ, 1 μM IVA) was applied for 2 days prior to the HRP assay (n = 10–12). Data represent mean values unless otherwise specified. Statistical significance was determined using one-way ANOVA followed by Dunnett's multiple comparison test (B and C) or a two-tailed unpaired t test (D, E, H, I). For data showing a significant effect, the corresponding p value is indicated in the figure.

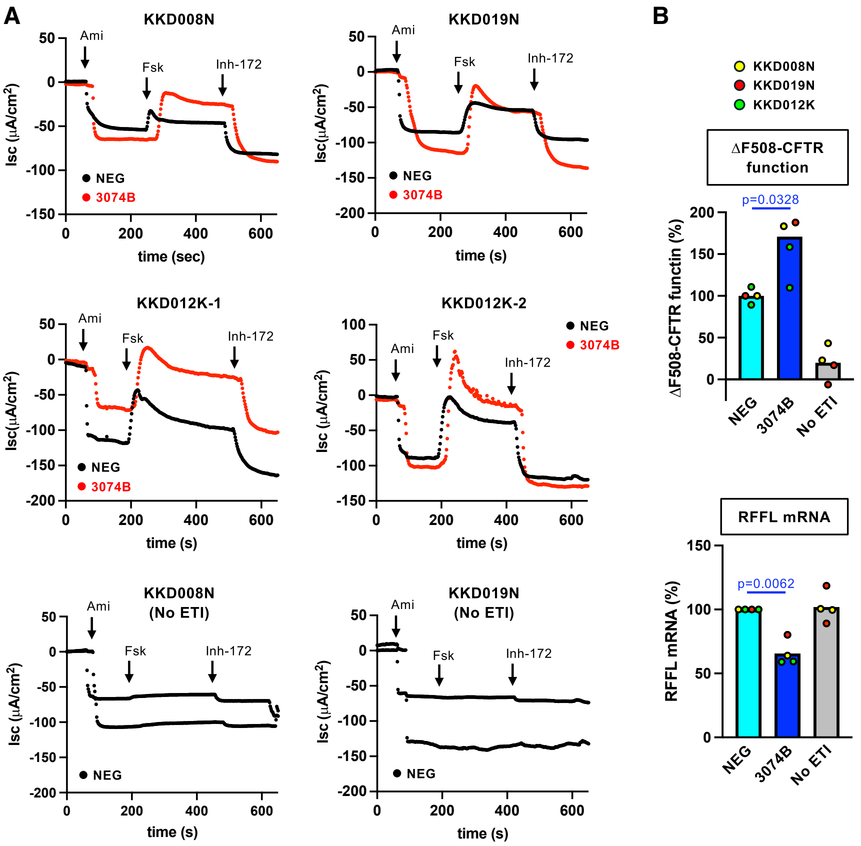

Figure 4. RFFL ASO 3074B enhances the efficacy of CFTR modulators in CF-HBE

(A) Effect of RFFL ASO 3074B (40 μM) in combination with ETI (3 μM ELX, 18 μM TEZ, 1 μM IVA) on endogenous CFTR function in primary CF-HBE cells homozygous for the ΔF508 mutation from three independent donors (KKD008N, KKD019N, KKD012K). CFTR-mediated Isc were stimulated by sequential acute addition of forskolin (Fsk, 10 μM) and subsequently inhibited by CFTRinh-172 (Inh-172, 20 μM). CF-HBE cells were pre-treated with negative control ASO (NEG) or RFFL ASO 3074B for 4 days and with ETI for the final 2 days. (B) Quantification of ΔF508-CFTR function from CFTRinh-172-sensitive Isc (A) in CF-HBE cells derived from three independent donors (KKD008N, KKD019N, KKD012K, upper). Untreated cells served as a negative control (no ETI). RFFL KD efficiency was assessed post hoc by qPCR (lower). Data represent mean values. Statistical significance was determined using a two-tailed paired Student's *t* test. For data showing a significant effect, the corresponding *p* value is indicated in the figure.

activity of rare CFTR mutants when combined with ETI treatment and may represent a promising therapeutic option for CF patients carrying these ultra-rare mutations.

DISCUSSION

Through the *de novo* design of ASOs and their evaluation across multiple cultured cell models, this study successfully developed an ASO that inhibits endogenous RFFL in bronchial epithelia derived from CF patients. While previous studies demonstrated that RFFL KD enhances the effect of CFTR modulators by preventing ΔF508-CFTR degradation at the PM in cell lines[10,11] and mouse organoids,[13] this study confirms the same effect in CF-HBE using RFFL ASO. Given that RFFL knockout mice exhibit no adverse phenotypes,[13,42] RFFL could be a promising drug target for CF therapy. Importantly, this ASO does not activate TLR9 even at high concentrations, indicating a low risk of triggering innate immune responses and supporting its favorable safety profile. Moreover, the ASO is effective in CF-HBE without the need for a transfection reagent and remains functional in a saline-compatible formulation, making it a strong candidate for future development as an inhalable spray directly targeting the airway epithelium, the primary site of CF pathology.[1] The increase in ΔF508-CFTR PM levels persisted for at least 6 days following a single ASO administration, suggesting the potential for reduced dosing frequency and improved treatment safety and convenience. This sustained effect is likely due to the high biostability conferred by the chemically modified nucleic acids used in the ASO.[43]

This study also demonstrates that RFFL ASO treatment potentiated the effects of ETI on CFTR mutants associated with rare forms of CF, including those with limited responsiveness to modulators, such as R560S-CFTR, R347P-CFTR, and A561E-CFTR. These findings suggest that *RFFL*-targeting ASOs represent a promising strategy to enhance ETI efficacy not only for the ΔF508 mutation but also across a broader spectrum of CFTR mutations. This conclusion is further supported by a strong correlation between the CFTR-rescuing effects of ETI alone and those observed with the combined ETI and RFFL ASO treatment (Figure 5C). Furthermore, treatment with RFFL ASO alone increased the PM level of class VI mutants such as T70-CFTR to levels comparable to those achieved with ETI treatment. This effect is likely due to inhibition of RFFL-mediated peripheral degradation of T70-CFTR, which is capable of reaching the PM but is unstable once there.[10,44] Similarly, RFFL ASO alone elevated the PM expression of the class II mutants R347P-CFTR and N1303K-CFTR.[45,46] Unlike T70-CFTR, which can partially localize to the PM,[44] R347P-CFTR is largely absent from the PM under physiological conditions (Figure S1). However, the observed increase in PM expression upon RFFL KD suggests that a small fraction of R347P-CFTR may reach the PM and be rapidly eliminated via the RFFL-mediated peripheral QC mechanism. Supporting this interpretation, a previous study detected a minor population of mature R347P-CFTR protein even in the absence of CFTR modulators.[45] Similarly, although not previously reported, RFFL may also be involved in the peripheral QC of N1303K-CFTR. While ASO 3074B enhanced the channel function of N1303K-CFTR and T70-CFTR in the presence of ETI,

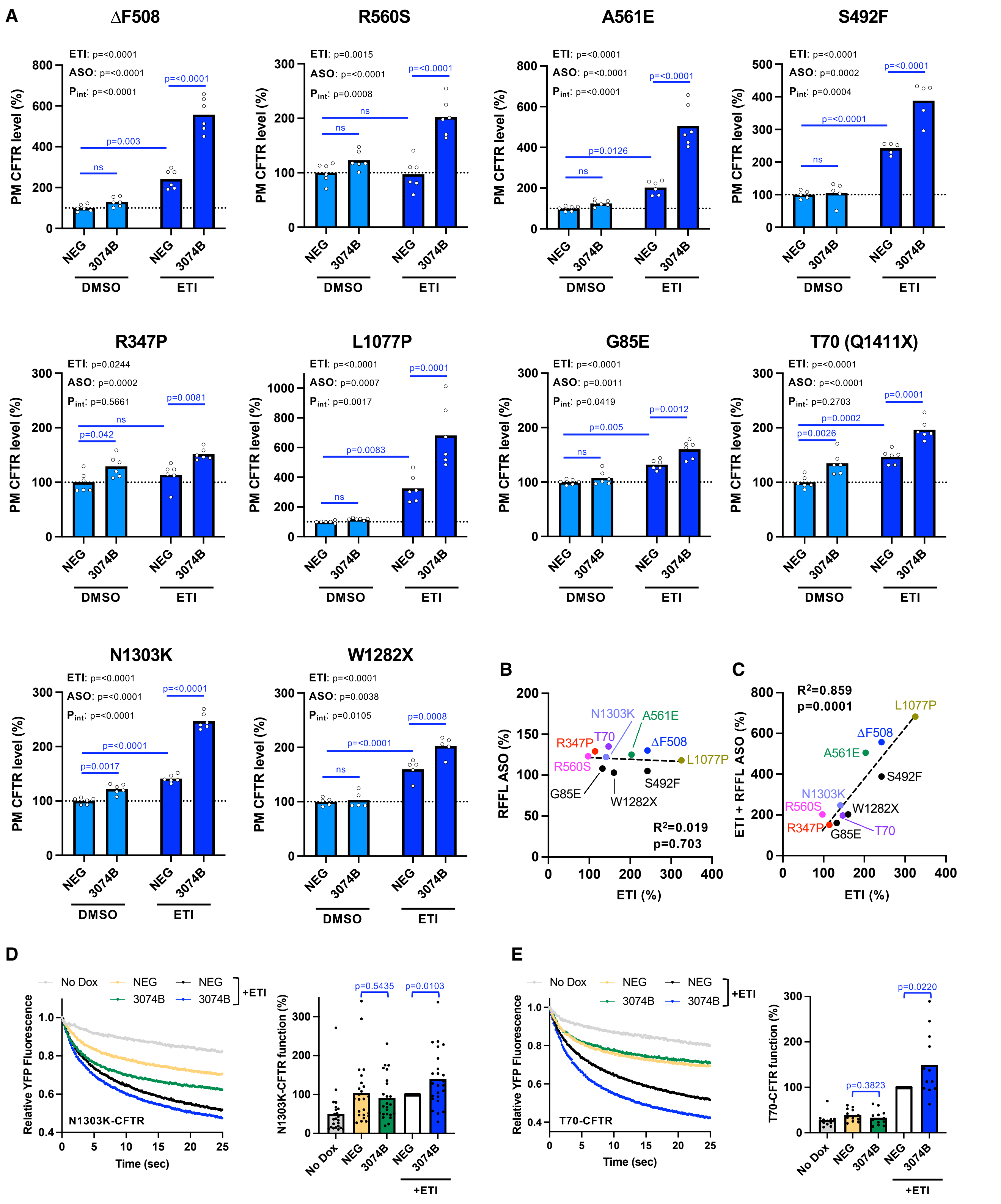

(legend on next page)

these improvements were not detected without ETI, likely due to the limited sensitivity of the YFP quenching assay. Future studies employing more sensitive evaluation methods will be needed to clarify whether ASO monotherapy can improve the function of these ultra-rare CFTR mutants.

Since RFFL ASO functions as a proteostasis regulator by targeting the peripheral protein QC mechanism, which is responsible for the degradation of various aberrant membrane proteins,[10] it may serve as a general stabilizer not only for CFTR but also for a wide range of membrane proteins associated with intractable diseases.[12,47] RFFL is overexpressed in certain types of cancers and cancer cell lines[48] and has been shown to promote cell migration[49] and proliferation.[50] Inhibition of RFFL expression results in the suppression of cancer cell growth[48] and the sensitizing of cancer cells to chemotherapeutic agents by regulating p53 levels.[51] Therefore, RFFL ASO may also be useful as a therapeutic agent for cancers involving high RFFL expression. Contrary to expectations,[51] RFFL ASO did not alter p53 levels in CFBE cells (Figure S3), suggesting that the contribution of RFFL to p53 regulation may be minimal in this context. Consistently, recent large-scale analyses of RFFL substrates have not reported p53 as being affected by RFFL knockout, further supporting the idea that RFFL plays only a limited role in regulating p53.[52] Instead, recent proteomic studies have identified novel RFFL substrates and implicated RFFL in fatty acid metabolism.[52] Although we cannot exclude the possibility that RFFL ASO could influence fatty acid metabolism in human tissues, the absence of abnormal phenotypes in RFFL knockout mice suggests that RFFL inhibition is unlikely to result in major adverse effects.

In summary, the *RFFL*-targeting ASO developed in this study represents a first-in-class CFTR stabilizer and a promising lead therapeutic candidate, with potential applicability not only to ΔF508-CFTR but also to rare CFTR mutations either in combination with ETI or as a standalone treatment.

MATERIALS AND METHODS

Synthesis of oligonucleotides

All RFFL ASOs and SY-ODN18 (Table 1) were synthesized and purified by Ajinomoto Bio-Pharma Services Japan GeneDesign, Inc. (Osaka, Japan).

Cell culture and transfection

HeLa, 293MSR, and CFBE Tet-on cells stably expressing ΔF508-CFTR-HRP (CFBE-ΔF508-CFTR-HRP) or ΔF508-CFTR-3HA (CFBE-ΔF508-CFTR-3HA) and BEAS-2B cells were cultured as previously described.[10,32,41,53] HeLa cells (Cat# TKG 0331) were obtained from the Cell Resource Center for Biomedical Research, Institute of Development, Aging and Cancer, Tohoku University (Japan). CFBE-ΔF508-CFTR-HRP and CFBE-ΔF508-CFTR-3HA cells were kindly provided by Dr. Gergely Lukacs (McGill University). 293MSR cells (Cat# R79507) and BEAS-2B cells (Cat# EC951 02433) were purchased from Thermo Fisher Scientific and KAC Co., Ltd. (Kyoto, Japan), respectively. BEAS-2B cells stably expressing CFTR variants with an extracellularly fused HiBiT tag and CFBE Tet-on cells stably expressing HBH-N1303K-CFTR-3HA were established via lentiviral transduction, as previously reported.[53] Recombinant lentiviruses were generated using the pLX304 destination vector system (Addgene #25890).

ASO (100 nM) transfection in HeLa, 293MSR, and CFBE cells was performed using the CEM method, as previously described.[31] When specified, lipofection was used for CFBE and BEAS-2B cells with Lipofectamine RNAiMAX (Thermo Fisher Scientific, Waltham, MA), following the manufacturer's protocol.

RT-qPCR assay for RFFL ASO knockdown efficiency

HeLa, 293MSR, and CFBE cells were transfected with 100 nM RFFL ASO using the CEM method, as previously described.[31] Briefly, ASO was added to culture medium containing 9 mM $CaCl_2$, and cells were incubated at 37°C for 24 h. Total RNA was then extracted, and endogenous *RFFL* mRNA levels were quantified by RT-qPCR. Results are expressed as the percentage of *RFFL* mRNA relative to control and are presented as a heatmap. For lipofection in CFBE cells, 20 nM ASO was used.

Total RNA was extracted from cells in 24-well plates using TRIzol Reagent (Thermo Fisher Scientific) according to the manufacturer's protocol. For reverse transcription, 500 ng of total RNA was used with ReverTra Ace qPCR RT Master Mix (TOYOBO, Osaka, Japan). RT-qPCR was carried out using the LightCycler 480 System (Roche Diagnostics) with SYBR Advantage qPCR Premix (TOYOBO). The expression level of *RFFL* mRNA was normalized to human GAPDH mRNA as an internal control and expressed as relative

Figure 5. Effect of RFFL ASO on PM expression of rare CFTR mutants

(A) Effect of RFFL ASO 3074B on PM levels of various CFTR mutants tagged with extracellular HiBiT in BEAS-2B cells. Cells were transfected with 20 nM negative control ASO (NEG) or RFFL ASO 3074B (3074B) via lipofection. Four days post-transfection, PM expression of CFTR mutants was quantified (n = 5–6). Prior to analysis, cells were treated with either DMSO (0.3%) or ETI (1 μM ELX, 3 μM TEZ, 1 μM IVA) for 2 days at 37°C. Statistical significance was assessed using two-way ANOVA with Holm-Sidak's multiple comparisons test. Significant interaction effects are indicated as P_{int}. For data showing a significant effect, the corresponding p value is indicated in the figure. (B) Correlation between the effects of RFFL ASO and ETI on the PM levels of CFTR-HiBiT variants in BEAS-2B cells, as measured in (A). (C) Correlation between the effects of ETI alone and the combined treatment of ETI + RFFL ASO on the PM levels of CFTR-HiBiT variants in BEAS-2B cells, as measured in (A). Correlations were analyzed by linear regression, and the corresponding R^2 and p values are indicated. (D and E) The channel function of HBH-N1303K-CFTR-3HA (D, n = 24) and T70-CFTR-3HA (E, n = 12) in CFBE Tet-on cells transfected with 50 nM NEG or ASO 3074B was assessed using the halide-sensitive YFP quenching assay. CFTR expression was induced with doxycycline (1 μg/mL) for 4 days, and cells were pre-treated with ETI (1 μM ELX, 3 μM TEZ, 1 μM IVA) for 2 days at 37°C prior to the assay. CFTR channel activity was quantified as the initial YFP quenching rate. Data represent mean values. Statistical significance was determined using a two-tailed unpaired Student's t test.

Table 1. Oligonucleotide list

ASO #	Name	Sequence (5′→3′)
1	ASO-0221(15)	GGGTTGGAATAGGCC
2	ASO-0222(15)	AGGGTTGGAATAGGC
3	ASO-0975(15)	GGTCACTCTCTCCAT
4	ASO-0976(15)	GGGTCACTCTCTCCA
5	ASO-0977(15)	CGGGTCACTCTCTCC
6	ASO-0978(15)	CCGGGTCACTCTCTC
7	ASO-0979(15)	GCCGGGTCACTCTCT
8	ASO-0988(15)	CCTTGTATAGCCGGG
9	ASO-0989(15)	TCCTTGTATAGCCGG
10	ASO-1000(15)	GTCCTTTCTGATCCT
11	ASO-1001(15)	AGTCCTTTCTGATCC
12	ASO-1037(15)	CCGTTTTGGTCTTCG
13	ASO-1055(15)	CCTGATGGTACTGCT
14	ASO-1056(15)	GCCTGATGGTACTGC
15	ASO-1057(15)	AGCCTGATGGTACTG
16	ASO-1559(15)	ACAGGAGGAGGGGTG
17	ASO-1565(15)	TTGGGTACAGGAGGA
18	ASO-1636(15)	TGTGCTGGGGAGGAG
19	ASO-2916(15)	GCGTTTAGGGTTTGC
20	ASO-3074(15)	GGTTTAGTGGTTGGT
21	ASO-3075(15)	GGGTTTAGTGGTTGG
22	ASO-3076(15)	GGGGTTTAGTGGTTG
23	ASO-3077(15)	AGGGGTTTAGTGGTT
24	ASO-3078(15)	AAGGGGTTTAGTGGT
25	ASO-3181(15)	AAAGGCTGTGGTTCA
26	ASO-3198(15)	ATTCCCCCAACTCCT
27	ASO-6232(15)	ACCATTCAGCCTTGC
28	ASO-6237(15)	TAGGCACCATTCAGC
29	ASO-3070(15)	TAGTGGTTGGTGATG
30	ASO-3072(15)	TTTAGTGGTTGGTGA
31	ASO-3069(17)	TTAGTGGTTGGTGATGG
32	ASO-3071(17)	GTTTAGTGGTTGGTGAT
33	ASO-3073(17)	GGGTTTAGTGGTTGGTG
34	ASO-3075(17)	AGGGGTTTAGTGGTTGG
35	ASO-3077(17)	AAAGGGGTTTAGTGGTT
36	ASO-3074(15)-A	GGT*TTAGTGGTTGGT
37	ASO-3074(15)-B	GGT*TTAGTGGTT*GGT
38	ASO-3074(15)-C	GG*T*TTAGTGGTT*G*GT
NEG	NC-ASO	AAGGCTAGACAAAGG
SY-ODN18	SY-ODN18	TCGTTTTGTCGTTTTGTC

siRNA	Name	Cat number
siNT	siNT (AllStars Neg. Control siRNA)	Qiagen Cat # 1027281
siRFFL	siRFFL-1	Qiagen Cat # SI00148232

Underline, AmNA; C, AmNA-5-methylcytidine; *, phosphodiester bond; siNT, non-targeting siRNA; siRFFL, RFFL-targeting siRNA; siRNA, small interfering RNA.

fold change. PCR amplification was performed using a two-step protocol with the following conditions: pre-incubation at 95°C for 3 min, 40 cycles of amplification (95°C for 5 s and 60°C for 30 s), and melting curve analysis (95°C for 5 min, 60°C for 60 s, and 97°C for final melt). The following primers were used for RT-qPCR:

GAPDH FW: 5′-CATGAGAAGTATGACAACAGCCT-3′

GAPDH RV: 5′- AGTCCTTCCACGATACCAAAGT -3′

RFFL FW: 5′- CAGCCCAGGTTCAGGAGA -3′

RFFL RV: 5′- CCAGGTAGACGGGTTCCTC -3′.

Measurement of ΔF508-CFTR PM expression by HRP assay

The HRP-based assay was performed as previously described.[10] Briefly, CFBE-ΔF508-CFTR-HRP cells were transfected with 20 nM RFFL ASO in 96-well plates using Lipofectamine RNAiMAX (Thermo Fisher Scientific), following the manufacturer's protocol. Five days after transfection, PM expression of ΔF508-CFTR-HRP was quantified using the HRP assay. CFTR expression was induced by treating cells with 1 μg/mL doxycycline (Dox) at 37°C for 4 days. To enhance ΔF508-CFTR trafficking to the PM, cells were incubated at 26°C for 2 days, followed by a 1-h rewarming at 37°C. Alternately, cells were treated with ETI (1 μM ELX (VX-445, Selleck Chemicals, Houston, TX), 3 μM TEZ (VX-661, Selleck Chemicals), and 1 μM IVA (VX-770, ChemScene, Monmouth Junction, NJ) at 37°C for 2 days. HRP activity was detected using the SuperSignal West Pico PLUS Chemiluminescent Substrate (Thermo Fisher Scientific) and quantified with a plate reader (Varioskan, Thermo Fisher Scientific).

Evaluation of cytotoxicity by alamarBlue assay

Following HRP activity measurement, cells in 96-well plates were washed with PBS (−) and incubated in medium containing alamarBlue reagent (ThermoFisher Scientific) for 2 h at 37°C. Fluorescence was measured using a plate reader (Varioskan, ThermoFisher Scientific) to assess cell viability and cytotoxicity.

Measurement of cell surface stability of CFTR

The cell surface stability of ΔF508-CFTR-3HA in CFBE Tet-on cells cultured in 24-well plates was assessed by cell surface ELISA. CFTR expression was induced with 1 μg/mL Dox for 4 days, and surface expression was enhanced by ETI treatment (1 μM ELX, 3 μM TEZ, 1 μM IVA) for 2 days at 37°C prior to the assay. Cell surface CFTR stability was determined by quantifying the loss of surface-bound anti-hemagglutinin (HA) antibody (labeled on ice for 1 h) during a 2-h chase at 37°C in the absence of ETI, as detailed previously.[10,32]

TLR9 activity assay

As previously described,[36] HEK-Blue hTLR9 cells were seeded at a density of 5×10^4 cells per well onto 96-well plates (Corning, Corning, NY) in DMEM supplemented with HEK-Blue Detection

(Invivogen, San Diego, CA). The cells in three wells per condition were immediately stimulated with 3074-B or SY-ODN18, an 18-nucleotide PS oligodeoxynucleotide with sufficient TLR9-stimulatory activity identified in our previous study (positive control)[36] or water (negative control) for 18 h. SEAP activity in the media, indicative of NF-κB activity triggered by TLR9 activation, was then determined for each well by measuring the absorbance at 650 nm using a microplate reader, ARVO X3 (PerkinElmer, Shelton, CT, USA). The obtained TLR9 activity values (n = 3 wells) were normalized to the values of the negative control group and presented as mean ± SD. Cell viability was determined by WST-8 assay (Cell Counting Kit-8 [Dojindo, Kumamoto, Japan]) according to the manufacturer's protocol.

Western blot analysis of ΔF508-CFTR and RFFL in CFBE Tet-on cells

CFBE-ΔF508-CFTR-3HA cells were transfected with 20 nM RFFL ASO using Lipofectamine RNAiMAX Transfection Reagent (Thermo Fisher Scientific), following the manufacturer's instructions. Five days post-transfection, cells were lysed in RIPA buffer (20 mM Tris-HCl, pH 8.0; 150 mM NaCl; 0.1% SDS; 1% Triton X-100; 0.5% sodium deoxycholate; 5 μg/mL pepstatin; 5 μg/mL leupeptin; 1 mM PMSF) for protein extraction. ΔF508-CFTR expression was induced by treatment with 1 μg/mL Dox for 4 days, and cells were treated with ETI (3 μM ELX, 1 μM TEZ, 1 μM IVA) during the final 2 days at 37°C. Protein levels of ΔF508-CFTR-3HA and RFFL were analyzed by western blotting using anti-HA (16B12, BioLegend, San Diego, CA) and anti-RFFL (HPA019492, Sigma-Aldrich, St. Louis, MO) antibodies, respectively.

Halide-sensitive YFP quenching assay

CFBE Tet-on cells stably expressing ΔF508-CFTR-3HA or T70-CFTR-3HA together with YFP,[10] or CFBE Tet-on cells stably expressing HBH-N1303K-CFTR-3HA, were seeded into black 96-well plates at 2.5 × 10^4 cells per well and transfected with 50 nM ASO. CFTR expression was induced with Dox (1 μg/mL) for 4 days, followed by ETI treatment for 2 days to promote correction. For halide-sensitive YFP expression in HBH-N1303K-CFTR-3HA cells, adenovirus encoding the halide-sensitive YFP variant (F46L/H148Q/I152L) was introduced at an MOI of 5–20, as described previously.[54] Four days after ASO transfection and 2 days after Ad-YFP infection, cells were washed three times with PBS-chloride buffer (140 mM NaCl, 2.7 mM KCl, 8.1 mM Na_2HPO_4, 1.5 mM KH_2PO_4, 1.1 mM $MgCl_2$, 0.7 mM $CaCl_2$, and 5 mM glucose). Each well was incubated with 50 μL of PBS-chloride, followed by addition of 50 μL of activator solution (20 μM forskolin, 0.5 mM IBMX, 0.5 mM cpt-cAMP, and 0.1 mM genistein) and incubated for 57 s. Fluorescence was continuously recorded at 200 ms intervals for 3 s (baseline) and for 32 s after rapid addition of 100 μL PBS-iodide (NaCl replaced with NaI). Measurements were obtained using a VICTOR Nivo multimode microplate reader (PerkinElmer) equipped with a dual syringe pump (excitation/emission: 500/535 nm). The rate of iodide influx was calculated by fitting the YFP fluorescence decay curve with GraphPad Prism 8 (GraphPad Software, San Diego, CA).

CF-HBE culture and ASO transduction

Primary cultured HBE (CF-HBE) cells from CF patients were purchased from the University of North Carolina at Chapel Hill, MLI Tissue Procurement and Cell Culture Core Facility. The CF-HBE cells were derived from three individuals homozygous for the ΔF508 mutation: KKD008N (age 27, female), KKD019N (age 38, female), and KKD012K (age 38, female). Cells were conditionally reprogrammed and grown in propagation medium described previously.[36,55] CF-HBE cells were grown at ALI on Snapwell filter supports (0.4 μm pore size, polyester, Corning) pre-coated with human placental collagen type VI (Sigma-Aldrich), and differentiated in differentiation medium[37,56] by approximately 1 month culture following established protocols.[37,56]

ASO transduction was performed in differentiated CF-HBE cells grown on Snapwell filter inserts following approximately 1 month of ALI culture. To remove the mucous layer, the apical surface was washed with 250 μL of PBS containing 3 mM dithiothreitol (FUJIFILM Wako Pure Chemical, Osaka, Japan) for 30 min, 4 days prior to transduction. An additional PBS wash was performed the day before transduction. ASO delivery was conducted via free uptake using a hypo-osmotic method as previously described.[57] Briefly, cells were incubated apically with 40 μM ASO in 250 μL of 50% PBS (hypo-osmotic solution) for 1 h at 37°C. The solution was then removed and replaced with 250 μL of 40 μM ASO in PBS. After 4 days, the apical medium was completely absorbed, and CFTR chloride channel function was assessed using the short-circuit current assay. ETI (3 μM ELX, 18 μM TEZ, and 1 μM IVA) was applied from the basolateral side for 2 days at 37°C prior to short-circuit current measurement, under conditions similar to those reported in a previous study.[9]

Measurement of endogenous ΔF508-CFTR activity by short-circuit current

Differentiated CF-HBE cells cultured on Snapwell inserts were mounted in Ussing chambers (U-2500, Warner Instruments, Hamden, CT) filled with Krebs-Bicarbonate Ringer (KBR) buffer on both the apical and basolateral sides. The transepithelial resistance of differentiated CF-HBE was >600 Ω/cm^2. The KBR buffer contained 115 mM NaCl, 19.8 mM $NaHCO_3$, 5.2 mM $KHCO_3$, 2.4 mM Na_2HPO_4, 0.4 mM NaH_2PO_4, 1.2 mM $CaCl_2$, 1.2 mM $MgCl_2$, and 5 mM glucose. The pH was maintained at 7.4 by continuous bubbling with a gas mixture of 95% O_2 and 5% CO_2. To generate a chloride ion gradient, the apical chamber buffer was replaced with Cl^--free KBR in which NaCl was substituted with 115 mM sodium D-gluconate.

Short-circuit current (I_{SC}) recordings were performed at 37°C using a CEZ-9100 measurement system (Nihon Kohden, Tokyo, Japan) connected to a PowerLab 2/26 data acquisition system (ADInstruments, Dunedin, NZ). To inhibit epithelial sodium channel (ENaC)

activity, 100 μM amiloride (TCI, Tokyo, Japan) was added to the apical chamber, and the current was monitored for 3–4 min until a stable inhibition plateau was reached. Subsequently, 10 μM forskolin (FUJIFILM Wako Chemicals) was added apically to stimulate CFTR-mediated Cl^- secretion, and the current was recorded for an additional 4–5 min until the response stabilized. Finally, 20 μM CFTR inhibitor-172 (Selleck Chemicals) was added to the apical side to inhibit CFTR, and the current was recorded for another 4–5 min. The forskolin-stimulated current inhibited by CFTR inhibitor-172 was quantified as the CFTR-specific Cl^- current.

Measurement of ΔF508-CFTR PM expression by HiBiT assay

BEAS-2B cells stably expressing CFTR-HiBiT (extracellular tag) were seeded in 96-well plates and transfected with 20 nM RFFL ASO using Lipofectamine RNAiMAX (ThermoFisher Scientific) according to the manufacturer's protocol. To enhance CFTR expression, cells were treated with 2 mM sodium butyrate for 2 days at 37°C. Where indicated, cells were also treated with ETI (1 μM ELX, 3 μM TEZ, 1 μM IVA) for 2 days at 37°C. Four days after ASO transfection, PM levels of CFTR-HiBiT were quantified using the Nano-Glo HiBiT Extracellular Detection System (Promega, Madison, WI) and measured using either a Luminoskan or Varioskan Flash microplate reader (Thermo Fisher Scientific).

Statistical analysis

Quantification was performed using data from a minimum of three independent experiments. Statistical significance was assessed using at least three biological replicates (n) through one- or two-way ANOVA or a two-tailed Student's *t* test, as specified in the figure legends, using GraphPad Prism 8. A *p* value of less than 0.05 was considered statistically significant. Although a normal distribution of the data was assumed, it was not formally tested.

DATA AVAILABILITY

The raw data required to reproduce these findings are available from the corresponding authors upon reasonable request.

ACKNOWLEDGMENTS

We thank D. Root (Broad Institute) for providing the pLX304 plasmid (Addgene #25890) and G. Lukacs (McGill University) for CFBE Tet-on cells stably expressing ΔF508-CFTR-HRP and ΔF508-CFTR-3HA. We also thank Y. Doi and H. Tateishi (Kwansei Gakuin University) for establishing BEAS-2B CFTR-HiBiT stable cell lines and for their technical support. This work was supported by JSPS KAKENHI (grants 19H05300 and 21H00294 to T.O.), the Kato Memorial Trust for Nambyo Research (to T.O.), and an Individual Special Research Subsidy with grants from Kwansei Gakuin University (to T.O.) and the Japan Agency for Medical Research and Development (AMED) under grant numbers JP21ae0121022 (to S.O.) and JP21ae0121024 (to T.I.).

AUTHOR CONTRIBUTIONS

T.O. contributed to the conception and design of the study. Y. Kasahara, S.O., T.Y., and T.I. contributed to the design and synthesis of the ASO. D.H., Y. Kai., R.F., and K.S. contributed to data acquisition. D.H. and T.O. performed statistical analysis and data interpretation and drafted the manuscript. Y. Kamada and T.O. reviewed and revised the manuscript.

DECLARATION OF INTERESTS

Y. Kasahara, S.O., T.I., and T.O. are applicants on a patent related to RFFL ASO (PCT/JP2025/030608). The other authors declare no competing interests.

DECLARATION OF GENERATIVE AI AND AI-ASSISTED TECHNOLOGIES IN THE WRITING PROCESS

During the preparation of this work the authors used ChatGPT in order to improve language and readability. After using this tool/service, the authors reviewed and edited the content as needed and take full responsibility for the content of the publication.

SUPPLEMENTAL INFORMATION

Supplemental information can be found online at https://doi.org/10.1016/j.omtn.2025.102756.

REFERENCES

1. Ong, T., and Ramsey, B.W. (2023). Cystic Fibrosis: A Review. JAMA *329*, 1859–1871. https://doi.org/10.1001/jama.2023.8120.
2. Riordan, J.R. (2008). CFTR function and prospects for therapy. Annu. Rev. Biochem. *77*, 701–726. https://doi.org/10.1146/annurev.biochem.75.103004.142532.
3. Ward, C.L., Omura, S., and Kopito, R.R. (1995). Degradation of CFTR by the ubiquitin-proteasome pathway. Cell *83*, 121–127.
4. Jensen, T.J., Loo, M.A., Pind, S., Williams, D.B., Goldberg, A.L., and Riordan, J.R. (1995). Multiple proteolytic systems, including the proteasome, contribute to CFTR processing. Cell *83*, 129–135. https://doi.org/10.1016/0092-8674(95)90241-4.
5. Bardin, E., Pastor, A., Semeraro, M., Golec, A., Hayes, K., Chevalier, B., Berhal, F., Prestat, G., Hinzpeter, A., Gravier-Pelletier, C., et al. (2021). Modulators of CFTR. Updates on clinical development and future directions. Eur. J. Med. Chem. *213*, 113195. https://doi.org/10.1016/j.ejmech.2021.113195.
6. Laselva, O., Guerra, L., Castellani, S., Favia, M., Di Gioia, S., and Conese, M. (2022). Small-molecule drugs for cystic fibrosis: Where are we now? Pulm. Pharmacol. Ther. *72*, 102098. https://doi.org/10.1016/j.pupt.2021.102098.
7. Wang, X., Tse, C., and Singh, A. (2025). Discovery and Development of CFTR Modulators for the Treatment of Cystic Fibrosis. J. Med. Chem. *68*, 2255–2300. https://doi.org/10.1021/acs.jmedchem.4c02547.
8. Zaher, A., ElSaygh, J., Elsori, D., ElSaygh, H., and Sanni, A. (2021). A Review of Trikafta: Triple Cystic Fibrosis Transmembrane Conductance Regulator (CFTR) Modulator Therapy. Cureus *13*, e16144. https://doi.org/10.7759/cureus.16144.
9. Keating, D., Marigowda, G., Burr, L., Daines, C., Mall, M.A., McKone, E.F., Ramsey, B.W., Rowe, S.M., Sass, L.A., Tullis, E., et al. (2018). VX-445-Tezacaftor-Ivacaftor in Patients with Cystic Fibrosis and One or Two Phe508del Alleles. N. Engl. J. Med. *379*, 1612–1620. https://doi.org/10.1056/NEJMoa1807120.
10. Okiyoneda, T., Veit, G., Sakai, R., Aki, M., Fujihara, T., Higashi, M., Susuki-Miyata, S., Miyata, M., Fukuda, N., Yoshida, A., et al. (2018). Chaperone-Independent Peripheral Quality Control of CFTR by RFFL E3 Ligase. Dev. Cell *44*, 694–708.e7. https://doi.org/10.1016/j.devcel.2018.02.001.
11. Taniguchi, S., Ito, Y., Kiritani, H., Maruo, A., Sakai, R., Ono, Y., Fukuda, R., and Okiyoneda, T. (2022). The Ubiquitin Ligase RNF34 Participates in the Peripheral Quality Control of CFTR (RNF34 Role in CFTR PeriQC). Front. Mol. Biosci. *9*, 840649. https://doi.org/10.3389/fmolb.2022.840649.
12. Fukuda, R., and Okiyoneda, T. (2018). Peripheral Protein Quality Control as a Novel Drug Target for CFTR Stabilizer. Front. Pharmacol. *9*, 1100. https://doi.org/10.3389/fphar.2018.01100.
13. Hinata, D., Fukuda, R., Ishiguro, H., Kamada, Y., and Okiyoneda, T. (2024). Enhanced CFTR modulator efficacy in ΔF508 CFTR mouse organoids by ablation of RFFL ubiquitin ligase. Biochem. Biophys. Res. Commun. *733*, 150433. https://doi.org/10.1016/j.bbrc.2024.150433.
14. Van Goor, F., Straley, K.S., Cao, D., González, J., Hadida, S., Hazlewood, A., Joubran, J., Knapp, T., Makings, L.R., Miller, M., et al. (2006). Rescue of DeltaF508-CFTR trafficking and gating in human cystic fibrosis airway primary cultures by small molecules. Am. J. Physiol. Lung Cell. Mol. Physiol. *290*, L1117–L1130. https://doi.org/10.1152/ajplung.00169.2005.

15. Awatade, N.T., Uliyakina, I., Farinha, C.M., Clarke, L.A., Mendes, K., Solé, A., Pastor, J., Ramos, M.M., and Amaral, M.D. (2015). Measurements of Functional Responses in Human Primary Lung Cells as a Basis for Personalized Therapy for Cystic Fibrosis. EBioMedicine *2*, 147–153. https://doi.org/10.1016/j.ebiom.2014.12.005.

16. Crooke, S.T., Baker, B.F., Crooke, R.M., and Liang, X.H. (2021). Antisense technology: an overview and prospectus. Nat. Rev. Drug Discov. *20*, 427–453. https://doi.org/10.1038/s41573-021-00162-z.

17. Islam, M.A., and Obika, S. (2023). Bridged Nucleic Acids for Therapeutic Oligonucleotides (Springer). https://doi.org/10.1007/978-981-19-9776-1_18.

18. Vinjamuri, B.P., Pan, J., and Peng, P. (2024). A Review on Commercial Oligonucleotide Drug Products. J. Pharm. Sci. *113*, 1749–1768. https://doi.org/10.1016/j.xphs.2024.04.021.

19. Stroes, E.S.G., Alexander, V.J., Karwatowska-Prokopczuk, E., Hegele, R.A., Arca, M., Ballantyne, C.M., Soran, H., Prohaska, T.A., Xia, S., Ginsberg, H.N., et al. (2024). Olezarsen, Acute Pancreatitis, and Familial Chylomicronemia Syndrome. N. Engl. J. Med. *390*, 1781–1792. https://doi.org/10.1056/NEJMoa2400201.

20. Yahara, A., Shrestha, A.R., Yamamoto, T., Hari, Y., Osawa, T., Yamaguchi, M., Nishida, M., Kodama, T., and Obika, S. (2012). Amido-bridged nucleic acids (AmNAs): synthesis, duplex stability, nuclease resistance, and in vitro antisense potency. Chembiochem *13*, 2513–2516. https://doi.org/10.1002/cbic.201200506.

21. Kanda, M., Kasahara, Y., Shimizu, D., Miwa, T., Umeda, S., Sawaki, K., Nakamura, S., Kodera, Y., and Obika, S. (2020). Amido-Bridged Nucleic Acid-Modified Antisense Oligonucleotides Targeting. Mol. Ther. Nucleic Acids *22*, 791–802. https://doi.org/10.1016/j.omtn.2020.10.001.

22. Uehara, T., Choong, C.J., Nakamori, M., Hayakawa, H., Nishiyama, K., Kasahara, Y., Baba, K., Nagata, T., Yokota, T., Tsuda, H., et al. (2019). Amido-bridged nucleic acid (AmNA)-modified antisense oligonucleotides targeting α-synuclein as a novel therapy for Parkinson's disease. Sci. Rep. *9*, 7567. https://doi.org/10.1038/s41598-019-43772-9.

23. Kanda, M., Kasahara, Y., Shimizu, D., Shinozuka, T., Sasahara, M., Nakamura, S., Iguchi, Y., Katsuno, M., Kodera, Y., and Obika, S. (2024). Dual-modified antisense oligonucleotides targeting oncogenic protocadherin to treat gastric cancer. Br. J. Cancer *131*, 1555–1566. https://doi.org/10.1038/s41416-024-02859-5.

24. Matsumoto, S., Yamamichi, T., Shinzawa, K., Kasahara, Y., Nojima, S., Kodama, T., Obika, S., Takehara, T., Morii, E., Okuyama, H., and Kikuchi, A. (2019). GREB1 induced by Wnt signaling promotes development of hepatoblastoma by suppressing TGFβ signaling. Nat. Commun. *10*, 3882. https://doi.org/10.1038/s41467-019-11533-x.

25. Shimojo, M., Kasahara, Y., Inoue, M., Tsunoda, S.I., Shudo, Y., Kurata, T., and Obika, S. (2019). A gapmer antisense oligonucleotide targeting SRRM4 is a novel therapeutic medicine for lung cancer. Sci. Rep. *9*, 7618. https://doi.org/10.1038/s41598-019-43100-1.

26. Hofacker, I.L. (2003). Vienna RNA secondary structure server. Nucleic Acids Res. *31*, 3429–3431. https://doi.org/10.1093/nar/gkg599.

27. Zuker, M. (2003). Mfold web server for nucleic acid folding and hybridization prediction. Nucleic Acids Res. *31*, 3406–3415. https://doi.org/10.1093/nar/gkg595.

28. Burdick, A.D., Sciabola, S., Mantena, S.R., Hollingshead, B.D., Stanton, R., Warneke, J.A., Zeng, M., Martsen, E., Medvedev, A., Makarov, S.S., et al. (2014). Sequence motifs associated with hepatotoxicity of locked nucleic acid–modified antisense oligonucleotides. Nucleic Acids Res. *42*, 4882–4891. https://doi.org/10.1093/nar/gku142.

29. Ohto, U., Shibata, T., Tanji, H., Ishida, H., Krayukhina, E., Uchiyama, S., Miyake, K., and Shimizu, T. (2015). Structural basis of CpG and inhibitory DNA recognition by Toll-like receptor 9. Nature *520*, 702–705. https://doi.org/10.1038/nature14138.

30. Yasuhara, H., Yoshida, T., Sasaki, K., Obika, S., and Inoue, T. (2022). Reduction of Off-Target Effects of Gapmer Antisense Oligonucleotides by Oligonucleotide Extension. Mol. Diagn. Ther. *26*, 117–127. https://doi.org/10.1007/s40291-021-00573-z.

31. Hori, S.I., Yamamoto, T., Waki, R., Wada, S., Wada, F., Noda, M., and Obika, S. (2015). Ca2+ enrichment in culture medium potentiates effect of oligonucleotides. Nucleic Acids Res. *43*, e128. https://doi.org/10.1093/nar/gkv626.

32. Okiyoneda, T., Barrière, H., Bagdány, M., Rabeh, W.M., Du, K., Höhfeld, J., Young, J.C., and Lukacs, G.L. (2010). Peripheral Protein Quality Control Removes Unfolded CFTR from the Plasma Membrane. Science *329*, 805–810. https://doi.org/10.1126/science.1191542.

33. Takeshita, F., Leifer, C.A., Gursel, I., Ishii, K.J., Takeshita, S., Gursel, M., and Klinman, D.M. (2001). Cutting edge: Role of Toll-like receptor 9 in CpG DNA-induced activation of human cells. J. Immunol. *167*, 3555–3558. https://doi.org/10.4049/jimmunol.167.7.3555.

34. Vollmer, J., Weeratna, R.D., Jurk, M., Samulowitz, U., McCluskie, M.J., Payette, P., Davis, H.L., Schetter, C., and Krieg, A.M. (2004). Oligodeoxynucleotides lacking CpG dinucleotides mediate Toll-like receptor 9 dependent T helper type 2 biased immune stimulation. Immunology *113*, 212–223. https://doi.org/10.1111/j.1365-2567.2004.01962.x.

35. Pollak, A.J., Cauntay, P., Machemer, T., Paz, S., Damle, S., Henry, S.P., and Burel, S.A. (2022). Inflammatory Non-CpG Antisense Oligonucleotides Are Signaling Through TLR9 in Human Burkitt Lymphoma B Bjab Cells. Nucleic Acid Ther. *32*, 473–485. https://doi.org/10.1089/nat.2022.0034.

36. Yoshida, T., Hagihara, T., Uchida, Y., Horiuchi, Y., Sasaki, K., Yamamoto, T., Yamashita, T., Goda, Y., Saito, Y., Yamaguchi, T., et al. (2024). Introduction of sugar-modified nucleotides into CpG-containing antisense oligonucleotides inhibits TLR9 activation. Sci. Rep. *14*, 11540. https://doi.org/10.1038/s41598-024-61666-3.

37. Veit, G., Xu, H., Dreano, E., Avramescu, R.G., Bagdany, M., Beitel, L.K., Roldan, A., Hancock, M.A., Lay, C., Li, W., et al. (2018). Structure-guided combination therapy to potently improve the function of mutant CFTRs. Nat. Med. *24*, 1732–1742. https://doi.org/10.1038/s41591-018-0200-x.

38. Lopes-Pacheco, M. (2016). CFTR Modulators: Shedding Light on Precision Medicine for Cystic Fibrosis. Front. Pharmacol. *7*, 275. https://doi.org/10.3389/fphar.2016.00275.

39. Veit, G., Roldan, A., Hancock, M.A., Da Fonte, D.F., Xu, H., Hussein, M., Frenkiel, S., Matouk, E., Velkov, T., and Lukacs, G.L. (2020). Allosteric folding correction of F508del and rare CFTR mutants by elexacaftor-tezacaftor-ivacaftor (Trikafta) combination. JCI Insight *5*, e139983. https://doi.org/10.1172/jci.insight.139983.

40. Borgo, C., D'Amore, C., Capurro, V., Tomati, V., Sondo, E., Cresta, F., Castellani, C., Pedemonte, N., and Salvi, M. (2022). Targeting the E1 ubiquitin-activating enzyme (UBA1) improves elexacaftor/tezacaftor/ivacaftor efficacy towards F508del and rare misfolded CFTR mutants. Cell. Mol. Life Sci. *79*, 192. https://doi.org/10.1007/s00018-022-04215-3.

41. Taniguchi, S., Ono, Y., Doi, Y., Taniguchi, S., Matsuura, Y., Iwasaki, A., Hirata, N., Fukuda, R., Inoue, K., Yamaguchi, M., et al. (2023). Identification of α-Tocopherol succinate as an RFFL-substrate interaction inhibitor inducing peripheral CFTR stabilization and apoptosis. Biochem. Pharmacol. *215*, 115730. https://doi.org/10.1016/j.bcp.2023.115730.

42. Ahmed, A.U., Moulin, M., Coumailleau, F., Wong, W.W.L., Miasari, M., Carter, H., Silke, J., Cohen-Tannoudji, M., Vince, J.E., and Vaux, D.L. (2009). CARP2 deficiency does not alter induction of NF-kappaB by TNFalpha. Curr. Biol. *19*, R15-R19–R17-19. https://doi.org/10.1016/j.cub.2008.11.040.

43. Takakusa, H., Iwazaki, N., Nishikawa, M., Yoshida, T., Obika, S., and Inoue, T. (2023). Drug Metabolism and Pharmacokinetics of Antisense Oligonucleotide Therapeutics: Typical Profiles, Evaluation Approaches, and Points to Consider Compared with Small Molecule Drugs. Nucleic Acid Ther. *33*, 83–94. https://doi.org/10.1089/nat.2022.0054.

44. Sharma, M., Pampinella, F., Nemes, C., Benharouga, M., So, J., Du, K., Bache, K.G., Papsin, B., Zerangue, N., Stenmark, H., and Lukacs, G.L. (2004). Misfolding diverts CFTR from recycling to degradation: quality control at early endosomes. J. Cell Biol. *164*, 923–933. https://doi.org/10.1083/jcb.200312018.

45. van Willigen, M., Vonk, A.M., Yeoh, H.Y., Kruisselbrink, E., Kleizen, B., van der Ent, C.K., Egmond, M.R., de Jonge, H.R., Braakman, I., Beekman, J.M., and van der Sluijs, P. (2019). Folding-function relationship of the most common cystic fibrosis-causing CFTR conductance mutants. Life Sci. Alliance *2*, e201800172. https://doi.org/10.26508/lsa.201800172.

46. Veit, G., Avramescu, R.G., Chiang, A.N., Houck, S.A., Cai, Z., Peters, K.W., Hong, J.S., Pollard, H.B., Guggino, W.B., Balch, W.E., et al. (2016). From CFTR biology toward combinatorial pharmacotherapy: expanded classification of cystic fibrosis mutations. Mol. Biol. Cell *27*, 424–433. https://doi.org/10.1091/mbc.E14-04-0935.

47. Okiyoneda, T., Apaja, P.M., and Lukacs, G.L. (2011). Protein quality control at the plasma membrane. Curr. Opin. Cell Biol. *23*, 483–491. https://doi.org/10.1016/j.ceb.2011.04.012.

48. McDonald, E.R., and El-Deiry, W.S. (2004). Suppression of caspase-8- and -10-associated RING proteins results in sensitization to death ligands and inhibition of tumor cell growth. Proc. Natl. Acad. Sci. USA *101*, 6170–6175. https://doi.org/10.1073/pnas.0307459101.

49. Gan, X., Wang, C., Patel, M., Kreutz, B., Zhou, M., Kozasa, T., and Wu, D. (2013). Different Raf protein kinases mediate different signaling pathways to stimulate E3 ligase RFFL gene expression in cell migration regulation. J. Biol. Chem. *288*, 33978–33984. https://doi.org/10.1074/jbc.M113.477406.

50. Dong, Y., Zhao, J., Wu, C.W., Zhang, L., Liu, X., Kang, W., Leung, W.W., Zhang, N., Chan, F.K.L., Sung, J.J.Y., et al. (2013). Tumor suppressor functions of miR-133a in colorectal cancer. Mol. Cancer Res. *11*, 1051–1060. https://doi.org/10.1158/1541-7786.MCR-13-0061.

51. Yang, W., Rozan, L.M., McDonald, E.R., Navaraj, A., Liu, J.J., Matthew, E.M., Wang, W., Dicker, D.T., and El-Deiry, W.S. (2007). CARPs are ubiquitin ligases that promote MDM2-independent p53 and phospho-p53ser20 degradation. J. Biol. Chem. *282*, 3273–3281. https://doi.org/10.1074/jbc.M610793200.

52. Narendradev, N.D., Marathe, S., Baboo, S., McClatchy, D.B., Diedrich, J.K., Jain, P., Purwar, R., Yates, J.R., and Srinivasula, S.M. (2025). Quantitative Proteomic Analysis Reveals JMJD6 and DNAJB11 as Endogenous Substrates of E3 Ligase RFFL. J. Proteome Res. *24*, 3913–3930. https://doi.org/10.1021/acs.jproteome.5c00086.

53. Taniguchi, S., Berenger, F., Doi, Y., Mimura, A., Yamanishi, Y., and Okiyoneda, T. (2024). Ligand-based virtual-screening identified a novel CFTR ligand which improves the defective cell surface expression of misfolded ABC transporters. Front. Pharmacol. *15*, 1370676. https://doi.org/10.3389/fphar.2024.1370676.

54. Hinata, D., Beppu, S., Kamada, Y., and Okiyoneda, T. (2025). RFFL inhibition increases cell surface CFTR and reduces IL-8 production in airway epithelial cells upon COPD-associated environmental pathogen exposure. Biochem. Biophys. Res. Commun. *777*, 152251. https://doi.org/10.1016/j.bbrc.2025.152251.

55. Liu, X., Ory, V., Chapman, S., Yuan, H., Albanese, C., Kallakury, B., Timofeeva, O.A., Nealon, C., Dakic, A., Simic, V., et al. (2012). ROCK inhibitor and feeder cells induce the conditional reprogramming of epithelial cells. Am. J. Pathol. *180*, 599–607. https://doi.org/10.1016/j.ajpath.2011.10.036.

56. Neuberger, T., Burton, B., Clark, H., and Van Goor, F. (2011). Use of primary cultures of human bronchial epithelial cells isolated from cystic fibrosis patients for the pre-clinical testing of CFTR modulators. Methods Mol. Biol. *741*, 39–54. https://doi.org/10.1007/978-1-61779-117-8_4.

57. Michaels, W.E., Bridges, R.J., and Hastings, M.L. (2020). Antisense oligonucleotide-mediated correction of CFTR splicing improves chloride secretion in cystic fibrosis patient-derived bronchial epithelial cells. Nucleic Acids Res. *48*, 7454–7467. https://doi.org/10.1093/nar/gkaa490.

OMTN, Volume *36*

Supplemental information

Antisense oligonucleotide targeting the E3 ligase RFFL potentiates CFTR modulator efficacy in CF primary bronchial epithelial cells

Daichi Hinata, Yukari Kai, Ryosuke Fukuda, Yuka Kamada, Yuuya Kasahara, Kiyomi Sasaki, Tokuyuki Yoshida, Satoshi Obika, Takao Inoue, and Tsukasa Okiyoneda

Supplemental Methods

CFTR-Nluc degradation assay

CFBE Tet-on ΔF508-CFTR-3HA-Nluc cells were generated by lentiviral transduction using the pLIX vector as previously described.[10] Cells were seeded in 96-well plates and transfected with 20 nM ASO using Lipofectamine RNAiMax. Analyses were performed 4 days after transfection. CFTR expression was induced with 1 µg/mL doxycycline (Dox) for 4 days, and cells were treated with ETI (1 µM ELX, 3 µM TEZ, 1 µM IVA) for 2 days at 37° C. To minimize the contribution of immature ΔF508-CFTR during Nano-Glo Endurazine substrate loading, cells were treated with 100 µg/mL cycloheximide (CHX) for 3 hours at 37° C in CO_2-independent medium (ThermoFisher) according to the manufacturer's instructions. Following 3 hours of substrate loading, ΔF508-CFTR-Nluc luminescence was recorded continuously (every 10 min) at 37° C using a Luminoskan microplate reader (ThermoFisher). The degradation rate of ΔF508-CFTR-Nluc was calculated by fitting the luminescence decay curve with a one-phase exponential decay function in GraphPad Prism 8 (GraphPad Software).

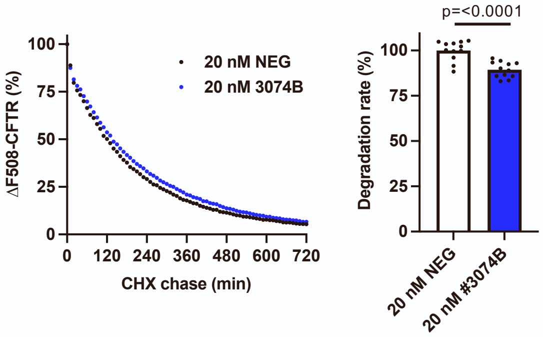

Figure S1.

Effect of RFFL ASO on mature ΔF508-CFTR-Nluc degradation in CFBE cells

Representative traces of ΔF508-CFTR-Nluc degradation in CFBE Tet-on cells transfected with 20 nM ASO are shown (left). Cells were pre-treated with ETI (1 µM ELX, 3 µM TEZ, 1 µM IVA) for 2 days at 37° C. To reduce the contribution of immature ΔF508-CFTR, cells were treated with CHX for 3 hours before measurement. The degradation rate of mature ΔF508-CFTR-Nluc was quantified (right). Statistical significance was assessed by unpaired t-test. Data represent mean values (n = 12).

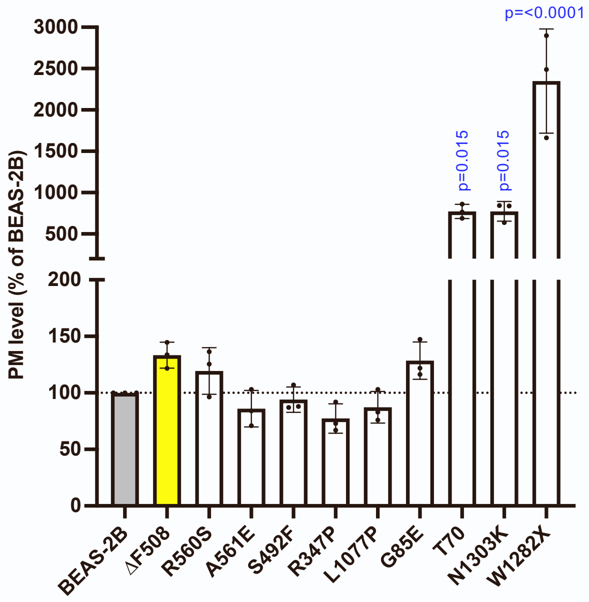

Figure S2.

PM levels of CFTR-HiBiT variants in BEAS-2B cells

Basal PM expression levels of CFTR-HiBiT mutants in BEAS-2B cells were quantified using the HiBiT assay and expressed as a percentage relative to parental BEAS-2B cells, which served as the background signal. Data are presented as mean ± SD (n = 3). Statistical significance was determined using one-way ANOVA followed by Dunnett's multiple comparison test. For data showing a significant effect, the corresponding *p*-value is indicated in the figure.

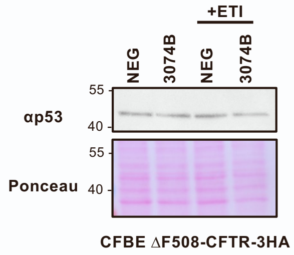

Figure S3.

Effect of RFFL ASO on p53 protein levels

Western blot analysis of ΔF508-CFTR-3HA in CFBE Tet-on ΔF508-CFTR-3HA cells transfected with 20 nM of either negative control ASO (NEG) or RFFL ASO 3074B (#37). Cells were treated with ETI (1 μM ELX, 3 μM TEZ, and 1 μM IVA) for 2 days at 37° C. The same lysates used in Fig. 3A were analyzed, and p53 was detected using an anti-p53 antibody (F16A10, Selleck, Cat#F0020). Ponceau staining was used as a loading control.
